# Retinoids Issued from Hepatic Stellate Cell Lipid Droplet Loss as Potential Signaling Molecules Orchestrating a Multicellular Liver Injury Response

**DOI:** 10.3390/cells7090137

**Published:** 2018-09-13

**Authors:** Marie Bobowski-Gerard, Francesco Paolo Zummo, Bart Staels, Philippe Lefebvre, Jérôme Eeckhoute

**Affiliations:** Institut Pasteur de Lille, The University of Lille, Inserm, CHU Lille, U1011-EGID, F-59000 Lille, France; marie.gerard@inserm.fr (M.B.-G.); francesco.zummo@inserm.fr (F.P.Z.); bart.staels@pasteur-lille.fr (B.S.); philippe-claude.lefebvre@inserm.fr (P.L.)

**Keywords:** liver, hepatic stellate cells, lipid droplet, retinoids, liver injury, intercellular communications

## Abstract

Hepatic stellate cells (HSCs) serve as the main body storage compartment for vitamin A through retinyl ester (RE)-filled lipid droplets (LDs). Upon liver injury, HSCs adopt a myofibroblastic phenotype characterized by an elevated expression of extracellular matrix proteins and a concomitant loss of LDs. On the one hand, LD breakdown has been suggested to provide the energy required for HSC activation into myofibroblast-like cells. On the other hand, this process could mitigate HSC activation following the transformation of released REs into retinoic acids (RAs), ligands for nuclear receptors exerting antifibrotic transcriptional regulatory activities in HSCs. Importantly, RAs may also constitute a means for HSCs to orchestrate the liver response to injury by triggering transcriptional effects in multiple additional surrounding liver cell populations. We envision that new approaches, such as single-cell technologies, will allow to better define how RAs are issued from LD loss in HSCs exert a multicellular control of the liver (patho)physiology.

## 1. Introduction

While the liver is composed mainly of hepatocytes, additional less abundant cell-types also contribute significantly to hepatic (patho) physiology. Hepatic stellate cells (HSCs), which are localized within the space of Disse between endothelial cells and hepatocytes, are mesenchymal-like cells making up about 10% of the total liver cell population [1]. Upon liver injury, HSCs undergo an “activation” process, described as a “transdifferentiation” towards a myofibroblast-like phenotype accompanied by a loss of the lipid droplets (LDs) characterizing their quiescent state [2]. Activated HSCs synthesize large amounts of ECM proteins, such as type I and type III collagen, and are therefore key players in the resolution of liver injury. However, uncontrolled HSC activation is a major contributor to liver fibrosis, which has put a spotlight on this cell type within the last decades [3].

## 2. Mechanisms of LD Loss and Requirement for HSC Activation

While the loss of LDs is a key feature of HSC activation, many questions remain as to how and why these LDs disappear from HSCs. Several pathways related to lipid metabolism have been suggested to account for LD loss upon activation. First, LD loss could be due to a modification of the activity of the enzymes involved in LD synthesis and/or degradation through pathways well known to be involved in the breakdown of adipose tissue LDs, including adipose triglyceride lipase/patatin-like phospholipase domain containing 2 (PNPLA2/ATGL) [4,5]. PNPLA3, whose expression is increased upon HSC activation, has also been shown to be involved in LD breakdown [6,7]. In addition, many recent studies have highlighted a role for autophagy in lipid release from HSCs that could help the myofibroblastic transition [3,8]. For instance, reactive oxygen species (ROS), produced by damaged hepatocytes, can induce autophagy through Rab25 and trigger LD disappearance in HSCs [9].

**Figure 1:**
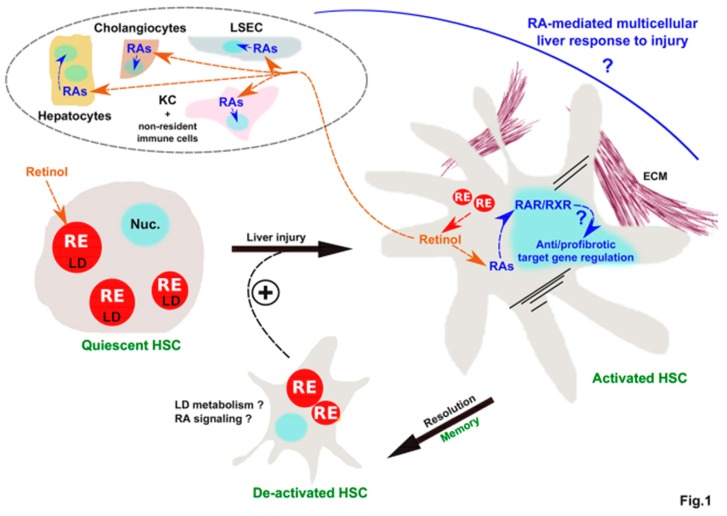
Potential role of RAs derived from the loss of RE-filled HSC LDs as an orchestrator of a multicellular response to liver injury. Schematic showing the basis for the proposal that RAs derived from the loss of RE-filled LD upon HSC activation may define a multicellular response to liver injury. Outstanding questions are indicated by question marks. See text for details. ECM, extracellular matrix; KC, Kupffer cells; LD, lipid droplet; LSEC, liver sinusoidal endothelial cells; Nuc., nucleus; RE, retinyl ester; RAs, retinoic acids.

It has been suggested that LD loss could support the HSC activation process essentially by providing energy (Figure 1). Indeed, the hydrolysis of triglycerides provides the cell with free fatty acids (FFAs) that can undergo mitochondrial β-oxidation to generate the ATP necessary for the acquisition of the myofibroblastic phenotype, including the production of α-sma and collagen fibers [10]. However, novel approaches to detect and quantify LDs showed that while activated HSCs isolated from bile duct ligation (BDL)-injured mice display smaller LDs in diameter, the number of LDs remains similar to healthy livers [11]. In line with this observation, lipidomic analysis revealed the apparition of “new” LDs in activated HSCs that contain less RE, but are enriched in triacylglycerol compared to the “original” LDs found in quiescent HSCs. These new LDs are thought to migrate to the cell periphery and to be smaller in size [12,13]. Moreover, LD REs are not lost until day 4 in spontaneously activated primary HSCs in vitro after transdifferentiation has already been initiated [11]. Finally, mice deficient for the *lecithin retinol acyltransferase* (*Lrat*) gene, encoding an enzyme converting retinol to its RE storage form [14], lack RE-filled LDs but are not predisposed to spontaneous or acute exacerbation of fibrogenesis, suggesting that the loss of LDs may not be sufficient to trigger liver fibrosis [14,15].

All together, these data indicate that energy provided by LD loss may not be required to initiate HSC activation, but may rather serve to sustain activation (perpetuation phase). Importantly, LDs in quiescent HSCs are filled with REs, which are released upon LD loss and can give rise to transcriptionally active retinoids. We postulate that this unique feature may represent a key signaling event within HSCs and surrounding liver cells upon the loss of RE-filled LD.

## 3. Control of HSC Activation through Retinoids Issued from LD Loss

Vitamin A must be acquired from the diet either as REs (animal origin) or provitamin A carotenoids (vegetal origin). Unlike most vitamins, retinol can be stored within the body at relatively high levels, probably to prevent vitamin A deficiency [16]. This storage occurs primarily in HSCs (~80% of total vitamin A) under the form of REs in LDs. This allows the liver to provide the peripheral tissues with sufficient vitamin A for months, even when dietary intake is not sufficient, through the release of retinol-bound retinol-binding protein (RBP) into the circulation. The rapid uptake of retinol by peripheral tissues is followed by its processing into active metabolites such as retinal or retinoic acids (RAs), which are instrumental to many processes including vision (pigment formation in the eyes), reproduction, growth, development, immunity and metabolism including adipose tissue metabolism [17]. Of note, many epidemiological studies have reported vitamin A deficiency in chronic liver diseases such as viral hepatitis and non-alcoholic fatty liver disease (NAFLD) [17,18]. For instance, an inverse correlation between liver retinol levels and the progression of NAFLD, as judged by histological classification of the disease, has been demonstrated [19] and could illustrate the loss of RE-filled LDs upon HSC activation. This leads to unbalanced retinol levels in the circulation and several organs.

Within HSCs, the retinol generated by RE hydrolysis can be metabolized into retinal through alcohol dehydrogenases (ADHs) and subsequently metabolized into RAs by retinal dehydrogenases (RALDHs) [20]. RAs derived from vitamin A can bind to the retinoic acid receptors (RARα, β and γ) and/or retinoid X receptors (RXRα, β, and γ), transcription factors (TFs) of the nuclear receptor (NR) superfamily. Importantly, RXRs can regulate the activity of various other NR family members by formation of heterodimers. Different studies using synthetic RAR/RXR ligands have been performed in order to monitor how these TFs can modulate HSC activation and gene expression. In rodents, RAs have been shown to exert antifibrotic activities [21,22,23]. This is in line with in vitro studies showing that the expression of pro-fibrogenic genes such as *collagen*, *type I*, *alpha 1* (*Col1a1*) or *actin*, *alpha 2*, *smooth muscle*, *aorta* (*Acta2* or *α-sma*) decreases upon exposure of HSCs to RAR/RXR activators [17,18]. Note, however, that specific effects are exerted by different RAs [24]. Moreover, while LRAT shows decreased expression/activity during HSC activation [15,25], RAs could oppositely increase *Lrat* mRNA expression through the presence of an RA responsive element in its promoter [16]. Indeed, inactivation of HSCs by RAs is associated with the recovery of large LDs, a feature of quiescent/de-activated HSCs. This may be due to both stimulation of LD synthesis and inhibition of their breakdown by autophagy; the latter involving modulation of LD-associated proteins [8]. Besides studies using synthetic RAs, indirect evidence that RAs modulate HSC activation also exists. For instance, the PNPLA3 I148M variant, found in NAFLD patients, leads to reduced retinol levels and RA signaling in HSC together with a profibrogenic phenotype [6]. Moreover, *Adh3* deficiency in the mouse exacerbates both liver fibrosis and the expression of profibrotic genes in HSCs [26].

All together, these observations strongly suggest that RAs release upon LD loss upon HSC activation could serve as a feedback loop mitigating HSC activation. This might be relevant to liver fibrosis resolution, which involves the reversion of a fraction of activated HSCs to a quiescent-like (de-activated) phenotype (Figure 1) [27,28]. However, the precise molecular mechanisms have not been investigated and whether the decreased expression of HSC activation markers results from direct transcriptional regulatory effects and/or indirectly results from modulation of LD metabolism remains to be defined.

## 4. Retinol Released by LD Loss as A Potential Cross-Talk Signal Between HSC and Other Hepatic Cell Populations in Liver (Patho) Physiology

Liver (patho)physiology relies on intercellular communications where the microenvironment is used to dialogue with surrounding cells [3,29,30]. While this has not yet been firmly established, we postulate that retinol release by HSC consecutive to LD loss may modulate activities of other cell types in the liver (Figure 1). Indeed, analyses of RAR/RXR cistromes and target genes in hepatocytes point to extensive crosstalk with key hepatic transcription factors in regulating both metabolic and housekeeping functions [31,32,33,34]. In line, synthetic RAs promote liver regeneration through induction of cell cycle gene expression in hepatocytes [35]. Further indications that endogenous RAs might be required for liver regeneration comes from *Lrat*^−/−^ mice, which lack HSC retinoid stores in LDs and show delayed regenerative responses upon partial hepatectomy [36]. Moreover, selective ablation of RXRα in hepatocytes impairs their proliferative and regenerative potential [37]. Importantly, RARs and RXRs are widely expressed in the liver, not only in HSCs and hepatocytes, but also in cholangiocytes, liver sinusoidal endothelial cells (LSEC), and Kupffer cells [38,39,40]. We propose that RAs may exert protective effects upon liver injuries also through activities in non-parenchymal cells. Interestingly, although still sparse, some studies reported functional RA signaling in liver non-parenchymal cells. In Kupffer cells, RXR activation might reduce nitric oxide and TNF-alpha production [41]. RAs regulate cholangiocyte proliferation in response to cholestatic injury [42] and in LSECs, which express functional RALDHs, RA signaling is required for liver priming of a CD4+ T cell gut-homing phenotype [43]. Finally, vitamin A plays an important role in both innate and adaptive immune functions. Thus, in addition to the production of cytokines, HSCs may modulate the liver immunology system through RAs issued from LD loss [44]. In line, HSC’s ability to produce RA is required to promote liver immune tolerance through promoting Tregs and suppressing Th17 differentiation [45].

Considering the biological effects of synthetic RAs on liver cell populations summarized here, the concept that retinol released by LD loss upon HSC activation might represent a protective mechanism is attractive. As retinol increases in the circulation following HSC activation, we envision that systemic effects may also occur.

## 5. Conclusions and Future Perspectives

While LD loss is the main feature of HSC activation, how this impacts on liver (patho)physiology remains a matter of debate. Indeed, LD loss might both fuel HSC activation by providing FFA-derived ATP and mitigate this process through RA signaling. How these two adverse consequences of LD loss concur to modulate HSC activation remains to be defined. RA signaling might, in addition, trigger protective responses in surrounding liver cells (Figure 1). Direct monitoring of RA signaling in the various liver cell populations upon injury and HSC activation might now be at reach using low/single-cell transcriptomic and cistromic analyses. Such investigations would undoubtedly help to clarify the potential role of RA signaling in the multicellular control of liver response to injury. Importantly, the consequences of disturbed liver retinol metabolism most probably differ in acute and chronic conditions, the second being potentially linked to vitamin A deficiency.

An important and still poorly defined feature of HSC is their potential heterogeneity. Indeed, this may translate to HSC subpopulations with different abilities to store RE in LD and to activate upon injury [46]. Here again, single-cell approaches might provide valuable insights. Interestingly, the reversal of HSC activation during liver fibrosis resolution gives rise to deactivated HSCs, which are different from quiescent HSCs as they keep the memory of previous activation and are more responsive to a second fibrogenic stimulus (Figure 1) [27,28]. Of interest would be to characterize LD metabolism and RA signaling in these deactivated HSCs to define how they relate to the memory effects leading to a higher responsiveness to recurring liver insult.
